# 

**Published:** 1911-10-07

**Authors:** 


### THE HOUSING QUESTION.

Last year we drew attention to the arrangements made by the Building and Estates Development Company, Limited, of Bristol, whereby doctors were offered a country house on Calway's patent reinforced monolithic concrete-wall system, with cavities in the walls, for about £450. We now understand that many doctors have availed themselves of the opportunity thus presented to build on a thoroughly reliable and at the same time strikingly economical plan, and that they have expressed their satisfaction with the result. In fact, the Company has been so inundated with orders that it has only been able to undertake a third of the work offered this year. This illustrates the advisability on the part of intending builders of consulting the plans offered by the Company at the earliest opportunity. We have no hesitation in recommending the professional man, or the institutional worker, who desires to build his own house, to make a trial of the Calway system. The walls of these reinforced-concrete houses are much stronger than those built in brickwork, while they can be constructed at a cost of 25 per cent. lower. The designs of the houses are picturesque: the structures are absolutely dry and are ready for occupation on the day the building is finished, while they are extremely suitable for extreme or variable climates. Anyone who intends to build should make a point of writing to the firm and procuring a copy of "Picturesque Homes," which contains all information regarding the system. Our advertisement columns this week supply some further data of interest concerning these houses.

